# A direct nuclear magnetic resonance method to investigate lysine acetylation of intrinsically disordered proteins

**DOI:** 10.3389/fmolb.2022.1074743

**Published:** 2023-01-06

**Authors:** Olivia A. Fraser, Sophia M. Dewing, Emery T. Usher, Christy George, Scott A. Showalter

**Affiliations:** ^1^ Center for Eukaryotic Gene Regulation, Department of Biochemistry and Molecular Biology, The Pennsylvania State University, University Park, PA, United States; ^2^ Department of Chemistry, The Pennsylvania State University, University Park, PA, United States

**Keywords:** acetylation, nuclear magnetic resonance, histone, post-translational modification, intrinsically disordered protein

## Abstract

Intrinsically disordered proteins are frequent targets for functional regulation through post-translational modification due to their high accessibility to modifying enzymes and the strong influence of changes in primary structure on their chemical properties. While lysine N_ε_-acetylation was first observed as a common modification of histone tails, proteomic data suggest that lysine acetylation is ubiquitous among both nuclear and cytosolic proteins. However, compared with our biophysical understanding of the other common post-translational modifications, mechanistic studies to document how lysine N_ε_-acetyl marks are placed, utilized to transduce signals, and eliminated when signals need to be turned off, have not kept pace with proteomic discoveries. Herein we report a nuclear magnetic resonance method to monitor N_ε_-lysine acetylation through enzymatic installation of a^13^C-acetyl probe on a protein substrate, followed by detection through ^13^C direct-detect spectroscopy. We demonstrate the ease and utility of this method using histone H3 tail acetylation as a model. The clearest advantage to this method is that it requires no exogenous tags that would otherwise add steric bulk, change the chemical properties of the modified lysine, or generally interfere with downstream biochemical processes. The non-perturbing nature of this tagging method is beneficial for application in any system where changes to local structure and chemical properties beyond those imparted by lysine modification are unacceptable, including intrinsically disordered proteins, bromodomain containing protein complexes, and lysine deacetylase enzyme assays.

## 1 Introduction

**FIGURE 1:**
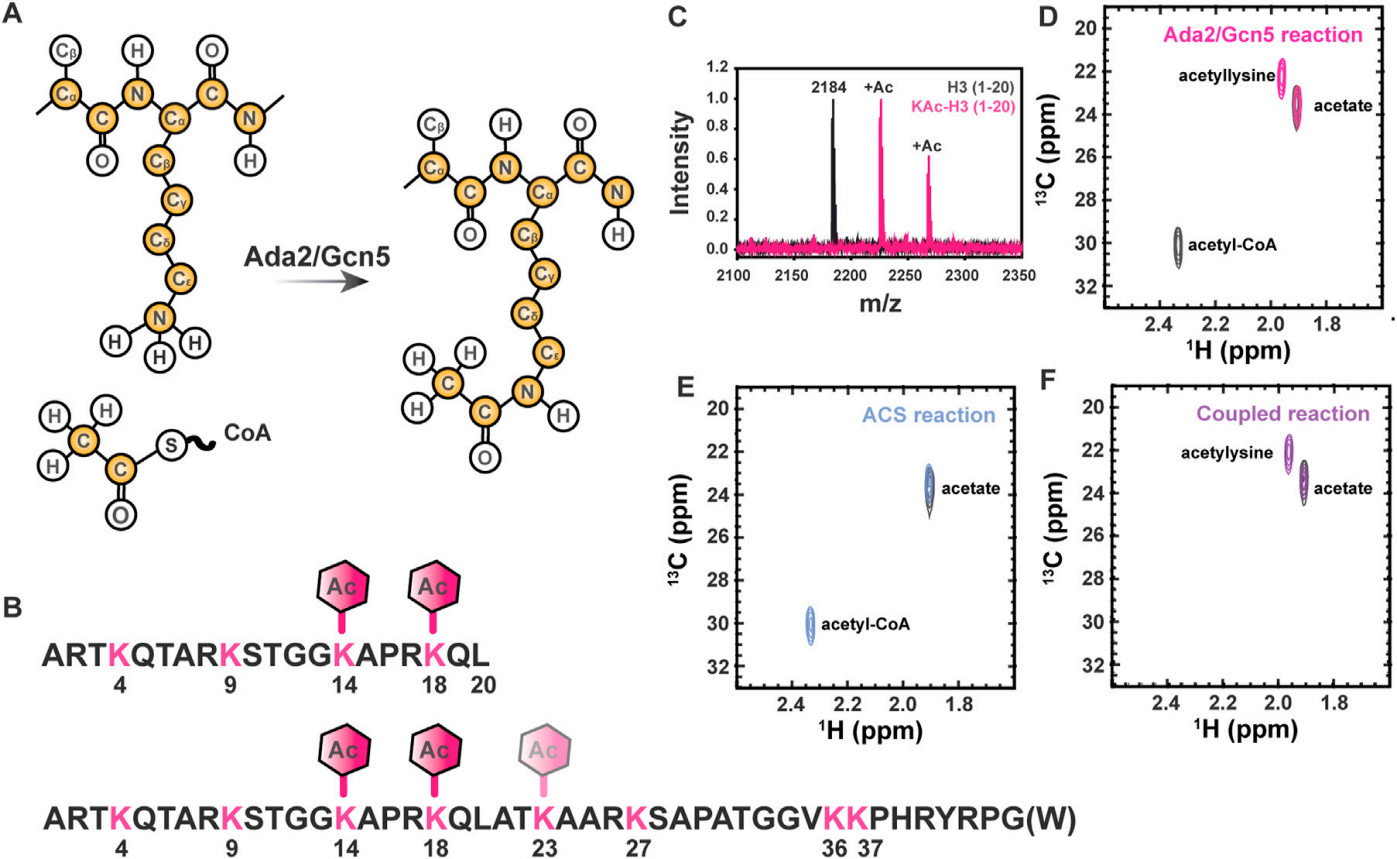
Lysine N_ε_ acetylation can be directly monitored by NMR spectroscopy if ^13^C-acetate is incorporated. **(A)** Lysine acetyltransferase (KAT) enzymes, such as Ada2/Gcn5 used in the current study, transfer ^13^C-enriched acetyl moieties to target proteins from ^13^C-labeled acetyl CoA molecules. Isotopically enriched atoms are shown in yellow. Note that ^15^N labeling of the target protein is optional and ^13^C labeling of the target protein is never required, but may be present in some samples as a result of other characterizations. **(B)** The sequence of the H3 (1-20) and H3 (1-44) constructs used for all NMR studies are shown in black. Lysines are shown in pink and expected acetylation events are annotated with hexagons. **(C)** MALDI-TOF mass spectrometry is used to observe acetylation of H3 (1-20). A control mass spectrum of H3 (1-20) before acetylation is shown in black. Two acetylation events can be seen in pink, corresponding to m/z of 2,225 and 2,368. **(D)** The [^1^H,^13^C]-HSQC spectrum of acetylated H3 1-20 (pink) displays a unique resonance corresponding to detection of acetyllysine. The free acetate peak is present in both the H3 spectrum the gray control spectrum without H3. Note the presence of the ^13^C-acetyl CoA resonance (2.32 ppm ^1^H, 30 ppm ^13^C) in the gray control spectrum. **(E)** Acetyl-CoA synthetase catalyzes the transfer of free ^13^C acetate to form ^13^C-acetyl-CoA (blue spectrum). The free ^13^C-acetate peak in a control spectrum (gray) prior to reaction is included for reference. **(F)** Coupling ACS-catalyzed generation of ^13^C-acetyl-CoA to Ada2/Gcn5-catalyzed generation of ^13^C-acetyllsine in H3 (purple spectrum) is highly efficient. Note the absence of accumulated ^13^C-acetyl-CoA. The free ^13^C-acetate peak in a control spectrum (gray) prior to reaction is included for reference.

Reversible chemical modification of proteins through the introduction of post-translational modifications (PTMs) is an important biological mechanism for control of protein function. Intrinsically disordered proteins (IDPs) exhibit enhanced accessibility of modifiable residues in comparison with even the surfaces and loops of structured proteins. Due to the lack of stable tertiary structure to impart functional specificity, the chemical properties imparted by the primary structure of IDPs, including charge and local structures imparted by intramolecular interactions, are extremely important to their function (Bah and Forman-Kay, 2016). The influence of PTM-state on these properties allows for dynamic changes to the behavior of IDPs. For example, lysine N_ε_ acetylation results in both changes to the availability of hydrogen bonding functional groups and charge neutralization of the sidechain (Figure 1A).

Acetylation can modulate protein stability, activity, and complex assembly as well as interact with other PTMs to regulate cellular and developmental processes such signal transduction, cell cycle programming, and gene expression (Narita et al., 2019). Lysine Nε acetylation was first discovered to occur on disordered N-terminal tails of histone proteins (Allfrey et al., 1964), where it has been associated with increased chromatin accessibility (Shogren-Knaak et al., 2006). Lysine N_ε_ acetylation has since been found to occur on thousands of non-histone proteins including p53 (Sakaguchi et al., 1998; Liu et al., 1999), α-tubulin (L’hernault and Rosenbaum, 1985), and NF-κB (Huang et al., 2010). Thus, methods to characterize the varying molecular mechanisms whereby acetylation impacts local protein structure and biomolecular interactions are imperative due to the high biological prevalence of this regulatory mark.

Despite the urgency of better characterizing lysine N_ε_ acetylation, protein acetylation is currently difficult to study on a biophysical level due to experimental limitations. Biochemically, the installation of the acetyl group can be monitored using a radioisotope labeled source of acetyl coenzyme A (acetyl-CoA). The use of ^3^H or ^14^C labeled acetyl-CoA provides the benefit of high sensitivity and does not require the use of an exogenous tag but is encumbered by the standard limitations of working with radioactive materials (Allfrey et al., 1964; Nelson et al., 1980; Ait-Si-Ali et al., 1998). Mass spectrometry provides crucial insights into the site specificity of lysine acetylation, and in the proteomic assays that led to the observation of widespread acetylation in the proteome (Hebert et al., 2013; Silva et al., 2013); yet, mass spectrometry is a destructive technique, which can limit its applicability. The downstream impact of acetylation can be studied using techniques such as mutagenesis to glutamine or the use of an acetyllysine mimic, such as the introduction of a thiocarbamate modification (Huang et al., 2010). These techniques may not be applicable in cases where reversibility of the lysine residue’s acetylation status is biologically necessary. Further, non-native modifications have the potential to yield artifacts due to the impact of changing the chemistry of the sidechain (Nagaraj et al., 2012). To study deacetylation, peptide substrates have been conjugated to fluorescent dyes such as 7-amino-4-methylcoumarin, but this impacts deacetylase activity (Toro et al., 2017). Thus, it is desirable to develop a label-free, site specific, and non-destructive technique to monitor lysine N_ε_ acetylation, readout by downstream proteins or nucleic acids, and reversibility through enzymatic deacetylation.

Nuclear magnetic resonance (NMR) is an advantageous method for studying PTMs of IDPs as it is label-free and non-destructive (Liokatis et al., 2010; Theillet et al., 2012). Conventional heteronuclear 2D NMR, such as the [^1^H, ^15^N]—HSQC, yields two observables that have been used to monitor acetylation events using ^15^N-labeled substrate. Firstly, whereas rapid exchange of the proton with solvent eliminates the lysine sidechain ^1^H_ε_-^15^N_ε_ resonances from most spectra, this NMR resonance becomes observable upon acetylation, due to the accompanying reduced rate of solvent exchange. Secondly, small changes in backbone chemical shift are often observable upon sidechain acetylation (Liokatis et al., 2010), yielding an indirect readout. These methods have a downside of requiring at the minimum ^15^N enriched peptide substrate. To fully benefit from site resolution, these experiments require a full determination of backbone chemical shift assignments. Thus, available techniques are characterized by relatively high cost in materials and time and require expertise that is often beyond many laboratories that might be interested in pursuing these studies.

The [^1^H,^15^N]-HSQC experiments described above have traditionally been performed without isotopic enrichment of the acetyl group transferred to the substrate protein, yet there are major advantages to be gained by instead transferring a^13^C-acetyl group for direct observation. Previously, we have developed a strategy analogous to this using a^13^C-SAM methyl donor to provide an NMR visible signal to monitor lysine methylation events (Usher et al., 2021). Here we demonstrate enzymatic transfer of uniformly ^13^C-acetyl groups, either with or without concomitant isotopic enrichment of the substrate protein. As the acetyllysine moiety bears chemical similarity to the peptide backbone, we hypothesized that modifying ^13^C direct-detect NMR methods typically used for observation of IDP backbone residues would provide an unambiguous and easily interpreted signal corresponding to an acetylation event. Thus, we report here modifications to the ^13^C direct-detect CACO and CON experiments that yield optimized detection of the acetamide functional group created upon lysine N_ε_ acetylation. This technique has the capability to dramatically advance knowledge of structure and function impacts from protein acetylation.

## 2 Materials and methods

Implementing the method described here relies on the enzymatic installation of a^13^C-enriched acetyl group to a peptide construct by a lysine acetyltransferase enzyme. To maximize utility, we provide two methods to produce ^13^C-acetyl CoA, an example protocol for preparation of NMR-quantity IDP, protocols for the production of a lysine acetyltransferase enzyme, and detailed protocols for appropriately implementing our NMR method. To demonstrate the utility of the methods on synthesized, natural abundance isotope peptide, we perform select experiments on H3 1-20 (Genscript). Unless use of this synthetic peptide is noted in the text, all reported spectra were collected on recombinantly expressed histone H3 (1-44).

### 2.1 Synthesis of ^13^C-Acetyl coenzyme A

#### 2.1.1 Organic

CoA lithium salt (1 equivalent, CoALA Biosciences, AC02) and 1,1ʹ,2,2 ʹ -^13^C acetic anhydride (1.8 equivalents, Cambridge Isotope Laboratories, CLM-1161-1) were combined in 200 μl .5 M sodium bicarbonate in a capped recovery vessel and incubated with on ice for 45 min (Peter et al., 2016). The product was characterized by 1D ^1^H NMR (Supplementary Figure S1A) and stored at −80°C without further purification. A 200 μL reaction yielded approximately 13.4 mg of ^13^C acetyl CoA.

#### 2.1.2 Enzymatic

*Escherichia coli* acetyl-CoA synthetase (ACS) was purified as described in Section 2.2.3 and acetyl transfer reactions were carried out with 50 μM ACS in 300 μL of 100 mM Tris pH 7.8, 700 μM acetate (1 equivalent), 700 μM CoA-Li salt (1 equivalent), 7 mM ATP (10 equivalents), and 10 mM MgCl_2_ (14.2 equivalents). Reactions were allowed to proceed at room temperature overnight (18 h) prior to supplementation with 5% D_2_O and 500 μM DSS. The reaction products were verified by ^1^H 1D NMR (Supplementary Figure S1B). Under these conditions, ACS (specific activity not determined) alone scavenges only 36% of available acetate as activated acetyl-CoA. However, when coupled to the Ada2/Gcn5 acetyl transfer reaction (Section 2.3.2), the acetyl-CoA produced by ACS is sufficient to acetylate H3 (1-20) as efficiently as synthetically derived acetyl-CoA and with improved ^13^C atom economy.

### 2.2 Expression and purification of protein constructs

The suite of NMR experiments described here allows flexibility in preparation of the protein of interest. If the protein of interest cannot be generated through recombinant expression, or if avoidance of isotopic labeling is desirable for any other reason, detection of the acetyllysine moiety on natural abundance protein can be achieved through the ^13^C_aliphatic_-^13^C_carbonyl_ selective detected experiments. In principle, this advantage allows for measurements to be made on protein samples from diverse sources, such as mammalian cell culture, without the need for isotopic enrichment of the protein substrate. To enable use of the full suite of experiments, including ^13^C_carbonyl_-^15^N selective detection, ^15^N-enriched protein must be generated through recombinant expression in *E. coli* or other sources. If ^13^C/^15^N-enriched protein is generated, the triple quantum filtered methyl-selective experiment (Section 2.5.2) allows visualization of the acetyllysine moiety without background interference (*vide infra*).

As a test case, here we provide data acquired from acetylation of *Homo sapiens* Histone H3 tail (residues 1-44 or 1-20, as indicated). Our technique is dependent on generation of the acetyltransferase enzyme of interest and, if the biosynthetic route is employed, expression and purification of ACS as well. To demonstrate our technique, we have chosen to recombinantly express Ada2 and Gcn5 (Ada2/Gcn5), which together are core components of the SAGA complex. As our protocols for expression and purification deviate from literature precedent, all four are included here for completeness.

#### 2.2.1 H3 (1–44) + W45

The plasmid (pET3a) coding for the *H. sapiens* Histone H3 tail (residues 1-44) was a gift from the lab of Catherine Musselman, University of Colorado Anschutz (Morrison et al., 2018). The constructs used herein contained a C-terminal Trp residue for the purpose of quantification. BL21 (DE3) *Fhu2A* ^*-*^ *E. coli* transformed with the H3 (1-44) plasmid were grown in rich media (LB, BP9723) for mass spectrometry or NMR without isotopic enrichment of the protein. To generate proteins for NMR spectroscopy with isotopic protein enrichment, cells were grown in M9 minimal media supplemented with 1 g/L^15^N-ammonium chloride (Cambridge Isotope Laboratories, DNLM-8739-PK), 1X MEM vitamins (Corning), 1 mM MgSO_4_, and 1X trace metals (Teknova, T1001). For uniformly enriched proteins, the minimal media was supplemented with 2 g/L^13^C_6_-glucose (Cambridge Isotope Laboratories, CLM-1392-PK), otherwise 2 g/L of ^12^C_6_-glucose were added to the media. All cultures were grown in media containing 100 μg/mL ampicillin for selection.

The cultures were grown at 37 °C to an optical density at 600 nm (OD600) of between .9 - 1 and subsequently induced with .4 mM isopropyl β-D-1-thiogalactopyranoside (IPTG). Expression proceeded at 37°C for 3 h, after which cells were harvested by centrifugation at 3,900 *xg*. The pellet was washed with and stored in 20 mM Tris, 20 mM NaCl, 2 mM EDTA, pH 7.5 at −80 °C until purification.

The cells were lysed in 50 mM Tris pH 9.0, 100 mM NaCl with 1 mM phenylmethylsulfonyl fluoride and protease inhibitor cocktail set V, EDTA-free (Millipore Sigma) *via* sonication for three rounds of 1 min (50% duty cycle) with 50% amplitude. Lysed cells were cleared by centrifugation at 14,000 *xg* for 30 min. The clarified supernatant was brought to 80 °C in a water bath for 6 min and incubated on ice for 2 min. The product was clarified by centrifugation at 14,000 *xg* for 25 min. The supernatant was filtered with a 5 μm syringe filter before addition to an SP sepharose gravity column equilibrated with 50 mM Tris pH 9.0, 100 mM NaCl. The column was washed with two column volumes (CV) of 50 mM Tris pH 9.0, 100 mM NaCl, 3 M Urea followed by 50 mM Tris pH 9.0, 100 mM NaCl, 6 M Urea (2 CV). The protein was eluted from the column using H3 purification buffer with the addition of 6 M urea and 500—1,000 mM NaCl in 100 mM increments, where the 700 mM fraction contained the protein of interest. The elution fraction was confirmed by SDS-PAGE (Tris-Tricine, 16%) with both 2,2,2-trichloroethanol and Coomassie stain and subsequently exchanged using a 3 kDa MWCO Amicon centrifugal filter (UFC9003) to 50 mM potassium phosphate, pH 6.5, 150 mM potassium chloride. Quantitation of unlabeled protein was carried out by amide bond detection *via* FTIR or for labeled protein the absorbance of a dilution series at 280 nm. Expected yield from purification of protein recombinantly expressed in 1 L of minimal media is 1.5–2 mg.

#### 2.2.2 Ada2/Gcn5 acetyltransferase

Cultures of *E. coli* BL21 (DE3) *Fhu2A* ^*-*^ cells containing the pST44-HISMBPNyAda2t5-yGcn5t10x2 polycistronic expression construct (gift from Song Tan, Pennsylvania State University) were grown at 37°C in LB medium supplemented with 50 μg/mL ampicillin (Tan, 2001; Tan et al., 2005; Sun et al., 2018). The growth temperature was adjusted to 18 °C at an OD_600_ of .2. Expression was induced with .5 mM IPTG when the OD_600_ reached .8-1.0 and proceeded for 16–18 h at 18°C. The cells were harvested by centrifugation for 30 min at 3,900 *xg* at 4°C, washed with 20 mM Tris, 20 mM NaCl, 2 mM EDTA, pH 7.5, and stored at −80°C.

Cells containing recombinant protein were lysed in 50 mM sodium phosphate pH 7.0, 300 mM sodium chloride, 10 mM imidazole, 5 mM β-mercaptoethanol,1 mM phenylmethylsulfonyl fluoride. The cell lysate was cleared by centrifugation for 30 min at 14,000 *xg* and 4°C. The supernatant was filtered through a 1 μm syringe filter and passed over Ni^2+^-NTA resin (G-Biosciences). Bound protein was washed with 50 mM sodium phosphate pH 7.0, 300 mM sodium chloride, 10 mM imidazole, 5 mM β-mercaptoethanol (2 CV) and eluted with 50 mM sodium phosphate pH 7.0, 300 mM sodium chloride, 100 mM imidazole, 5 mM β-mercaptoethanol (1.5 CV). The maltose-binding protein (MBP) and 6x His tags were removed by adding recombinant 6x His-tagged tobacco etch virus (TEV) protease to the elution fraction and dialyzing against 50 mM sodium phosphate pH 7.0, 300 mM sodium chloride, 10 mM imidazole, 5 mM β-mercaptoethanol for 48 h at 4°C. The dialysate was passed over Ni^2+^-NTA resin and washed with 50 mM sodium phosphate pH 7.0, 300 mM sodium chloride, 10 mM imidazole, 5 mM β-mercaptoethanol (2 CV), where Ada2/Gcn5 was captured in the flow through. The flow through was dialyzed against 50 mM sodium phosphate pH 7.0, 10 mM imidazole, 5 mM β-mercaptoethanol and concentrated by spin centrifugation using a 3 kDa MWCO Amicon centrifugal filter and supplemented with 20% glycerol for storage at −80°C.

#### 2.2.3 Acetyl-CoA synthetase

The plasmid pETM11-ACS, which was deposited by Frank Schulz, was purchased from Addgene (Addgene plasmid # 108943; http://n2t.net/addgene:108943; RRID:Addgene_108,943). *E. coli* BL21 (DE3) *Fhu2A* ^*-*^ cells containing the pETM11-ACS expression construct were grown in Luria Broth supplemented with 1 mM MgCl_2_ and 50 μg/mL kanamycin to OD_600_ of .6–.8. Protein expression was induced with .5 mM IPTG for 16 h at 30°C. The cells were harvested by centrifugation for 30 min at 3,900 *xg* at 4°C, washed with 20 mM Tris, 20 mM NaCl, 2 mM EDTA, pH 7.5, and stored at −80 °C.

All ACS purification steps were performed on ice unless otherwise noted. Cells containing recombinant protein were lysed by sonication in 100 mM Tris-HCl pH 7.5, 150 mM NaCl, 10% glycerol supplemented with protease inhibitor cocktail set V, EDTA-free and .2 mg/mL lysozyme (Sigma 12650-88–3). The cell lysate was cleared by centrifugation for 30 min at 14,000 *xg* and 4°C. The supernatant was filtered through a 1 μm syringe filter and passed over Ni^2+^-NTA resin (G-Biosciences). Bound protein was washed with 100 mM Tris-HCl pH 7.5, 150 mM NaCl, 10% glycerol, 50 mM imidazole, and eluted with 100 mM Tris-HCl pH 7.5, 150 mM NaCl, 10% glycerol, 150 mM imidazole. The elution fraction was dialyzed against 100 mM Tris-HCl pH 7.8 for 16 h at 4°C. Protein was concentrated by spin centrifugation and stored at −80°C. A typical yield is approximately 100 mg.

#### 2.2.4 Gcn5 BRD

Cultures of *E. coli* BL21 (DE3) Fhu2A-cells transformed with the plasmid (pET49b+) coding for *S. cervisiae* Gcn5 residues 329–438 (Gcn5 BRD) were grown at 37°C in LB medium supplemented with 50 μg/mL kanamycin. Expression was induced with .5 mM IPTG at an OD600 of .6–.8 and proceeded for 3 h. The cells were harvested by centrifugation for 30 min at 3,900 *xg* at 4°C, washed with 20 mM Tris, 20 mM NaCl, 2 mM EDTA, pH 7.5, and stored at −80°C.

Cells containing recombinant protein were lysed in 50 mM Tris pH 7.5, 500 mM sodium chloride, 20 mM imidazole, 2 mM β-mercaptoethanol, 1 mM phenylmethylsulfonyl fluoride. The cell lysate was cleared by centrifugation for 30 min at 14,000 *xg* and 4°C. The supernatant was filtered through a 1 μm syringe filter and passed over Ni^2+^-NTA resin (G-Biosciences). Bound protein was washed with 50 mM Tris pH 7.5, 500 mM sodium chloride, 20 mM imidazole, 2 mM β-mercaptoethanol, .1% Triton-X (5 CV) and eluted with 50 mM Tris pH 7.5, 500 mM sodium chloride, 200 mM imidazole, 2 mM β-mercaptoethanol (1.5 CV). The 6x His tag was removed by adding recombinant 6x His-tagged 3C-protease to the elution fraction and dialyzing against 50 mM Tris pH 7.5, 500 mM sodium chloride, 20 mM imidazole, 2 mM β-mercaptoethanol for 15 h at 4°C. The dialysate was passed over Ni^2+^-NTA resin and 50 mM Tris pH 7.5, 500 mM sodium chloride, 20 mM imidazole, 2 mM β-mercaptoethanol (2 CV), where Gcn5 BRD was captured in the flow through. The flow through was concentrated and passed over Sephacryl S-100 (Cytiva) in 50 mM Tris pH 7.5, 150 mM NaCl, 5 mM DTT. Fractions containing Gcn5 BRD were determined by absorbance at 280 nm and SDS-PAGE. The sample was stored at −80 in 50 mM sodium phosphate pH 6.5, 50 mM NaCl. The sample was dialyzed to 50 mM potassium phosphate pH 7.2, 150 mM KCl before addition to histone 3 tail.

### 2.3 Acetyl transfer by Ada2/Gcn5

#### 2.3.1 Transfer from synthetically derived acetyl-CoA

Acetylation of 500 μM H3 1-44 tail was carried out in a 500 μL reaction by 2.6 μM Ada2/Gcn5 in 50 mM Tris/50 mM BisTris, 100 mM sodium acetate, 1 mM DTT, pH 7.5. ^13^C acetyl-CoA (1 mM) was added to initiate the reaction, which proceeded for 3 h at room temperature. Following the reaction period, the acetyltransferase enzyme was heat inactivated at 80°C for 10 min and pelleted by centrifugation at 14,000 *xg* for 5 min. The supernatant was decanted and buffer exchanged to 50 mM potassium phosphate, 150 mM potassium chloride, pH 6.5 using 3 kDa MWCO Amicon centrifugal filters. The sample was concentrated to 250 μL (final concentration of H3: 1 mM). For NMR sample preparation, D_2_O (5%) was added to the sample, which was transferred to a 5/3 mm NMR tube (New Era Enterprises, NE-H5/3-Br) before further analysis.

#### 2.3.2 Transfer from enzymatically derived acetyl-CoA

To validate Ada2/Gcn5 activity under ACS reaction conditions, acetylation of 350 μM H3 (1-20) (1 equivalent) was carried out in 300 μL by 150 nM Ada2/Gcn5 in 100 mM Tris pH 7.8, 700 μM synthetically derived ^13^C-acetyl-CoA (2 equivalents). ACS activity when provided with 1,2-^13^C acetate was also individually validated in a 300 μL reaction containing 50 μM ACS in 100 mM Tris pH 7.8, 700 μM ^13^C-acetate (1 equivalent), 700 μM CoA-Li salt (1 equivalent), 7 mM ATP (10 equivalents), and 10 mM MgCl_2_ (14.2 equivalents). Finally, acetylation of 350 μM H3 1-20 (1 equivalent) was carried out in a 300 μL coupled reaction with 150 nM Ada2/Gcn5 and 50 μM ACS in 100 mM Tris pH 7.8, 700 μM ^13^C-acetate (2 equivalents), 700 μM CoA-Li salt (2 equivalents), 7 mM ATP (20 equivalents), and 10 mM MgCl_2_ (28.4 equivalents). Reactions were allowed to proceed at room temperature overnight (18 h) prior to supplementation with 5% D_2_O and 500 μM DSS. The reaction products were verified by [^1^H, ^13^C]—HSQC NMR. When coupled, these reactions accomplish efficient acetylation of H3 (1-20) with improved atom economy compared to the synthetic route and provide the advantage of utilizing ^13^C-acetate ($25.16/mmol at time of writing), a significantly more cost-effective starting material than ^13^C-acetic anhydride ($164.79/mmol a time of writing).

### 2.4 Mass spectrometric methods

Matrix-assisted laser desorption/ionization-time-of-flight and tandem time-of-flight mass spectrometry was used to collect mass spectra on unacetylated H3 1-20 peptide and H3 1-20 peptide acetylated by synthesized ^12^C acetyl-CoA and Ada2/Gcn5 using an Ultraflextreme instrument (Bruker). Samples were prepared by desalting with Pierce C-18 Spin Columns (Thermo Fisher Scientific, 89,870) followed by evaporation of solvent with centrifugation at 1,400 rpm at 30°C under vacuum. Samples were resuspended in 30% aqueous ACN and mixed 1:1 with matrix solution (either 10 mg/mL 4-chloro-a-cyanocinnamic acid or10 mg/mL a-cyano-4-hydroxycinnamic acid in 50% aqueous ACN and 2.5% formic acid). Acetylated peptides were fragmented by MS/MS in LIFT mode. Expected peptide fragment masses were calculated using the MS-product mode in the Protein Prospector web tool. The theoretical peaks were assigned using an R-script to the experimental fragments corresponding to K14Ace, K18Ace, and diacetylated H3 peptide (script available on Github, https://github.com/smdewing/MALDI-lift).

### 2.5 NMR methods

#### 2.5.1 Validation of semi-synthetic and enzymatic Acetyl-CoA production

^1^H-1D with water suppression using excitation sculpting with gradients for ^12^C labeled acetyl CoA (Hwang and Shaka, 1995), or ^1^H-1D with garp decoupling applied on the ^13^C channel for ^13^C labeled acetyl CoA were collected on a Bruker Avance AVIII 500 with TCI Cryoprobe using 65 k data points, 16 scans, a sweep width of 16 ppm, and a spectral Center of 4.69 ppm (Supplementary Figure S1).

#### 2.5.2 General methods for the collection of acetyllysine spectra

With the exceptions noted above, all NMR experiments were conducted on a Bruker Avance NEO 600 MHz spectrometer equipped with a 5 mm TCI triple-resonance cryoprobe and a SampleJet autosampler [^1^H, ^13^C]—HSQC experiments were collected using the standard pulse program from the Bruker Topspin library, with 1,024 direct data points and 128 indirect data points, 16 scans, and a sweep width of 2.98 ppm with a Center of 2.95 ppm in the direct dimension and 70 ppm with a Center of 40 ppm in the indirect dimension. and a recycle delay of 1 s. Hard 90° pules on ^1^H and ^13^C, as well as appropriate decoupling pulses, should be calibrated as per standard protocols on user instruments. For the spectra represented here, the typical 90° ^1^H pulse was 8 μs and the typical ^13^C 90° pulse was 15 μs. All pulsed field gradients were applied for 1 m with a sine shape.

All ^13^C direct-detect spectra were designed around strategies similar to those found in the previously reported [^13^C,^13^C]-CACO and [^13^C,^15^N]-CON experiments (Bermel et al., 2005). In all cases, virtual decoupling in the ^13^C direct dimension was achieved through utilization of in-phase anti-phase (IPAP) spectra processed to yield virtual decoupling using the standard ‘splitcomb’ AU program distributed with the Bruker Topspin library (Duma et al., 2003). Protocols for implementation of the standard Bruker library versions of the [^13^C,^13^C]-CACO-IPAP and [^13^C,^15^N]-CON-IPAP are thoroughly described elsewhere (Bastidas et al., 2015). All required ^1^H and ^15^N pulses are standard hard pulses or composite pulse decoupling sequences. For the spectra represented here, the typical 90° ^1^H pulse was 8 μs and the typical ^15^N 90° pulse was 25 μs. All pulsed field gradients were applied for 1 m with a sine shape. All pulses applied on the ^13^C channel are shaped, frequency selective pulses. Here, where all spectra were collected at 14.0 T, 90° ^13^C band-selective pulses used the Q5_sebop shape (or its time-reversed equivalent as noted in timing diagrams) and the 180° band-selective pulses used the Q3_surbop shape with durations of 350 µμs and 270 µs, respectively. Adiabatic inversion during the nitrogen chemical shift labeling period was achieved through application of a 500-m CHIRP pulse with 60 Hz sweep and 25% smoothing (Bohlen and Bodenhausen, 1993). Note that for spectrometers operating at alternative magnetic field strengths, pulse timings will vary, and on older spectrometers the traditional Q5 and Q3 pulses may be required as substitution for those listed above (Emsley and Bodenhausen, 1992). The user is referred to their manufacturer documentation, or to previous protocols as appropriate (Cook et al., 2018).

We report three variants of the acetyllysine, ^13^C_aliphatic_-^13^C_carbonyl_ selective, experiment. Each of the reported [^13^Cʹ,^13^C^ali^]-CaliCO-Kac variants present identical spectral information but utilize three different excitation schemes that the user may choose between depending on their specific application and needs. Inspiration for the reported pulse programs comes from the protonless CACO-IPAP (Bermel et al., 2005) and the 3D HNCOCa and 3D HCOCa (Serber et al., 2000), respectively. The Supplementary Material S1 includes timing diagrams corresponding to the protonless [^13^Cʹ,^13^C^ali^]-CaliCO-Kac (Supplementary Figure S2), amide-start [^13^Cʹ,^13^C^ali^]-CaliCO-Kac (Supplementary Figure S3), and methyl proton-start [^13^Cʹ,^13^C^ali^]-CaliCO-Kac (Supplementary Figure S4). All reported [^13^Cʹ,^13^C^ali^]-CaliCO-Kac experiments were recorded with 1,024 direct data points and 128 indirect data points, 64 scans, and a sweep width of 20 ppm with a Center of 172 ppm in the direct dimension, and a sweep width of 60 ppm with a Center of 25 ppm in the indirect dimension.

We report four variants of the acetyllysine, ^13^C_carbonyl_-^15^N selective, experiment. Each of the reported [^13^C,^15^N]-CON-Kac variants utilize different excitation schemes that the user may choose between depending on their specific application and needs. Inspiration for the reported pulse programs comes from the protonless CON-IPAP (Bermel et al., 2005), HN-flip CON-IPAP (Bermel et al., 2009), (HACA)-CON-IPAP (Bertini et al., 2011), and (HACA)-CON-ALA (Schubert et al., 1999; Sahu et al., 2014). The Supplementary Material S1 includes timing diagrams corresponding to the protonless [^13^C,^15^N]-CON-Kac (Supplementary Figure S5), amide-start [^13^C,^15^N]-CON-Kac (Supplementary Figure S6), methyl proton-start [^13^C,^15^N]-CON-Kac (Supplementary Figure S7), and triple quantum filtered methyl-selective [^13^C,^15^N]-CON-Kac (Supplementary Figure S8). All reported [^13^C,15N]-CON-Kac experiments were recorded with 1,024 direct data points and 256 indirect data points, 32 scans, and a sweep width of 20 ppm with a Center of 172 ppm in the direct dimension, and a sweep width of 42 ppm with a Center of 127 ppm in the indirect dimension. For ^13^C^methyl^-selective pulses, the offset from the carrier position should be set to Center the pulses on 25 ppm. The ^1^J_CCO_ in the acetamide functional group was measured to be 41.6 Hz, resulting in a refocusing delay of 4.9 m for this coupling. For the triple quantum selective experiment, which suppresses resonances from the backbone in uniformly ^13^C,^15^N-enriched proteins, the delay for ^13^C^ali^-^13^Cʹ was further shortened to reduce the intensity of artifacts resulting from long-range coupling into the backbone, resulting in a delay of 5.0 m, as annotated in the Supplementary Figure S8 legend.

#### 2.5.3 H3 binding to Gcn5 BRD

NMR experiments to monitor Gcn5 bromodomain binding to H3 were conducted on a 11.7 T Bruker Avance-3 spectrometer equipped with a TCI-cryoprobe. ^1^H-methyl-start [^13^C,^13^C] CaliCO-Kac experiments were collected with 1,024 direct data points and 128 indirect data points, 16 scans, and a sweep width of 20 ppm with a Center of 172 ppm in the direct dimension and 80 ppm with a Center of 172 ppm in the indirect dimension, and a recycle delay of 1 s.

#### 2.5.4 Processing and analysis

On-instrument processing of all acetyllysine spectra was performed using Bruker Topspin V 4.0.5. Additionally, 3D spectra were acquired to assign the backbone of H3 using our standard suite of ^13^C direct-detect experiments (Sahu et al., 2014), with on-instrument processing using Bruker Topspin V 3.2.6. Viewing and processing for image generation of all spectra were carried out in NMRFAM-SPARKY (Lee et al., 2015).

## 3 Results

Control of chromatin structure, promoter and enhancer accessibility, and subsequent levels of transcription at targeted loci through acetylation of multiple lysine residues within the Histone H3 N-terminal tail is one of the most extensively characterized examples of biological regulation by this ubiquitous post-translational mark (Tessarz and Kouzarides, 2014). Therefore, we have chosen to demonstrate the utility of our novel ^13^C direct-detect NMR strategy here using acetylation of H3 (1-44 or 1-20, as indicated) as a model system (Figure 1B). We first demonstrated acetylation of the disordered H3 1–20 by Ada2/Gcn5 using mass spectrometry for confirmation by means orthogonal to NMR. Two acetylation events were observed, corresponding to m/z of 2,225 and 2,268 (Figure 1C). The primary H3 lysine targeted by Gcn5 *in vitro* is K14 (Grant et al., 1999; Kuo and Andrews, 2013), although the Ada2/Gcn5 complex has previously been shown to also acetylate H3 at K18 *in vitro* (Balasubramanian et al., 2002). Acetylation of K14 and K18 was verified by MS/MS, thus authenticating our model system through established techniques.

Having verified that H3 (1-20) is acetylated on K14 and K18 by Ada2/Gcn5, we next collected NMR spectra of the modified proteins. Two methods were used to generate modified H3 1-20 tail: a two-step method relying on synthetic generation of ^13^C-acetyl CoA followed by acetyl-lysine modification of the H3 tail by Ada2/Gcn5, and a one-step method coupling enzymatic generation of ^13^C-acetyl CoA to acetyl-lysine modification of the H3 tail by Ada2/Gcn5. Control 1D spectra demonstrate successful transfer of acetate to CoA in both the organic and enzymatic routes (Supplementary Figure S1). [^1^H, ^13^C]—HSQC of the ACS reaction demonstrated transfer of ^13^C-acetate to generate ^13^C-acetyl-CoA (Figure 1D). Using synthetically or enzymatically derived ^13^C-acetyl CoA as a substrate, the [^1^H, ^13^C]—HSQC of ^12^C H3 yielded a new resonance at 1.87 ppm ^1^H, 22.0 ppm ^13^C, which is distinct from the resonances from free ^13^C acetate and ^13^C-acetyl CoA (Figures 1E,F). Thus, traditional ^1^H,^13^C-HSQC spectroscopy demonstrates the ease of detecting acetylated lysine residues by NMR.

**FIGURE 2:**
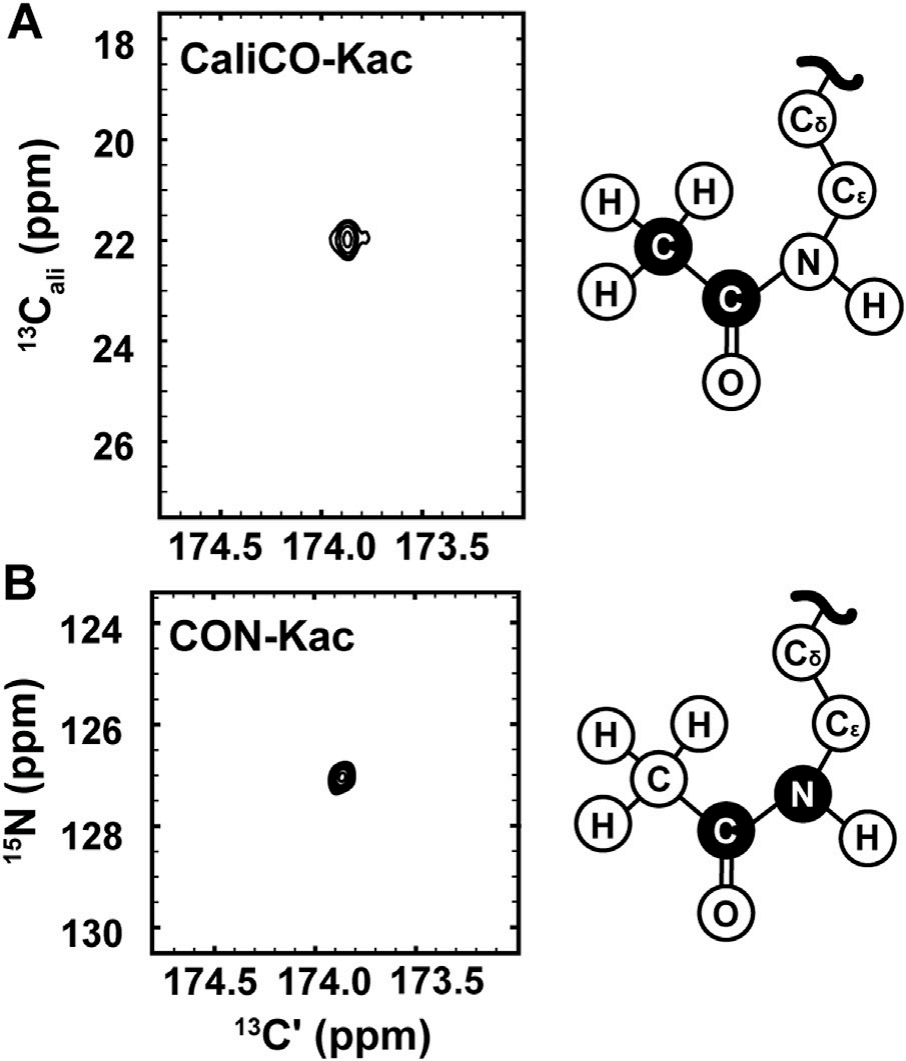
^13^C direct-detect NMR spectra recorded on acetyllysine residues from H3 (1-44) following transfer of ^13^C-acetyl by Ada2/Gcn5. **(A)** The [^13^Cʹ,^13^C^ali^] CaliCO-Kac contains a correlation between the carbonyl carbon and the methyl carbon of the acetyl group after enzymatic acetylation of H3 (orange spectrum). **(B)** The [^13^Cʹ,^15^N] CON-Kac contains a correlation between the carbonyl carbon and the N_ε_ of the acetamide functional group after enzymatic acetylation of H3. Structural depictions of the acetyllsine side chain are provided for reference, where the atoms contributing to the recorded resonances are colored black.

While the [^1^H, ^13^C]—HSQC spectrum has the advantage of a short collection time, under three minutes for the 350 μM H3 (1-20) samples reported here, ^13^C direct-detect NMR spectroscopy offers advantages for investigations of IDPs. For example, these spectra generally tolerate a wide range of solution conditions, including basic pH and high salt, that are often required to keep IDP samples soluble and stable, and are generally free from artifacts created in ^1^H-detected spectra by stabilizing co-solutes (Bastidas et al., 2015). Therefore, we set out to optimize acetyllysine-selective ^13^C direct-detect 2D spectra analogous to those employed with wide success in backbone-centric spectroscopy. For all spectra, the^13^C-acetyl group provides the source of ^13^C nuclei, while isotopic enrichment to produce ^15^N labeled H3 (1-44) peptide expands the suite of available experiments to include ^13^C-^15^N correlation spectroscopy. Following pulse program optimization, the [^13^C,^13^C] CaliCO-Kac (Figure 2A) and [^13^C,^15^N] CON-Kac (Figure 2B) both yield excellent spectral quality, with only a single major peak present, corresponding to the selected N_ε_ acetyllysine. Note that because mass spectrometry had already confirmed the presence of both K14 and K18 acetylation on H3 (1-20), this suggests that the chemical shifts of the H3 acetyllysine sites are degenerate.

**FIGURE 3:**
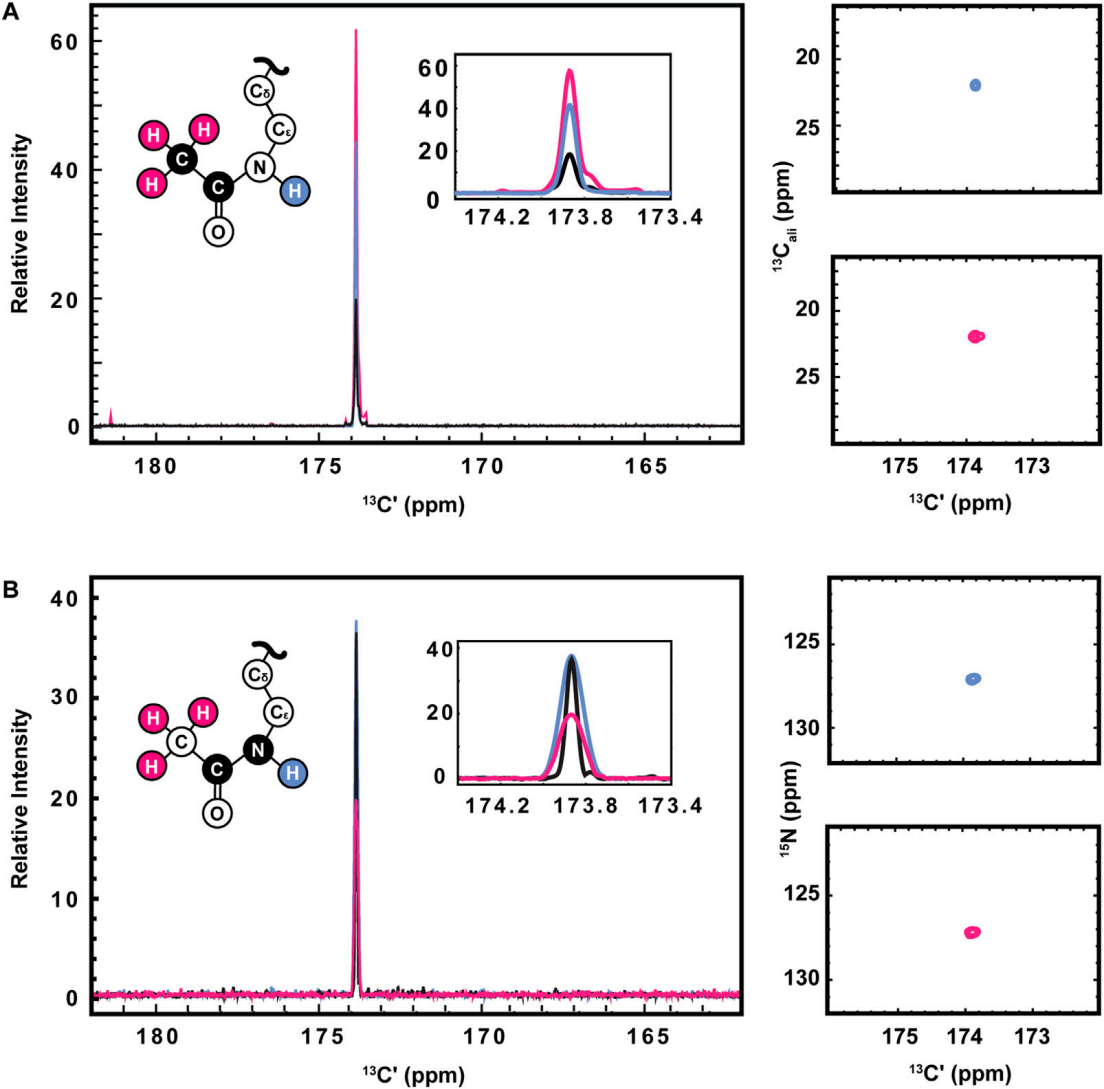
Optimization of ^1^H-start versions of the [^13^Cʹ,^13^C^ali^] CaliCO-Kac and [^13^Cʹ,^15^N] CON-Kac. **(A)** For the [^13^Cʹ,^13^C^ali^] CaliCO-Kac, ^1^H-start spectroscopy provides signal enhancement. ^13^C-start (black), ^1^H_N_-start (blue), and ^1^H_methyl_-start (pink) spectra are overlayed, displaying a clear benefit to initiating the magnetization transfer pathway from the ^1^H_methyl_ nuclei. **(B)** For the [^13^Cʹ,^15^N] CON-Kac, the ^1^H-start magnetization transfer pathway (black) displays the best combination of signal-to-noise and linewidth, while the ^1^H_N_-start (blue) approach provides no advantages, and the ^1^H_methyl_-start (pink) signal-to-noise is reduced. For both sets of spectra, zoomed in presentations of the 2D correlations from ^1^H_N_-start (blue), and ^1^H_methyl_-start spectra are displayed to the right of the 1D projections. Structural depictions of the acetyllsine sidechain are color coded to indicate the origin of magnetization in each of the three experimental variants.

^13^C direct-detect spectroscopy is well known to require higher sample concentrations than ^1^H detect methods, creating a burden on investigators who wish to leverage the other inherent advantages of this technique. For example, the data presented here were collected on samples of 800–1,000 μM H3 (1-44). Therefore, having demonstrated the utility of the [^13^C,^13^C] CaliCO-Kac and [^13^C,^15^N] CON-Kac experiments, we next aimed to increase their sensitivity by starting the magnetization transfer pathway on either the methyl or amide protons, instead of using the “protonless” ^13^C-start strategy of the foundational pulse sequences. For the [^13^C,^13^C] CaliCO-Kac, starting the magnetization transfer pathway from the methyl protons, followed by transfer to the methyl ^13^C, provided a 4-fold sensitivity enhancement for otherwise identical experimental conditions (Figure 3A). For samples that have been uniformly ^15^N-enriched, beginning the magnetization transfer pathway on the amide proton, followed by transfer into the carbonyl carbon for indirect chemical shift labeling is also an option. While this technique did yield a 2.5-fold sensitivity increase relative to an identically collected ^13^C-start spectrum, the amide-start version perhaps unsurprisingly underperformed the enhancement observed in the methyl-start experiment. In summary, the improved sensitivity of the ^1^H-methyl-start [^13^C,^13^C] CaliCO-Kac should be advantageous for applications to future proteins with more strict solubility limits.

Having demonstrated improved [^13^C,^13^C] CaliCO-Kac in the proton-start formats tested, we next set out to optimize the [^13^C,^15^N] CON-Kac. Surprisingly, no increase in the signal-to-noise per scan was noted in the ^1^H-amide-start [^13^C,^15^N] CON-Kac experiment, while the sensitivity of the ^1^H-methyl-start experiment was diminished 2-fold relative to ^13^C-start (Figure 3B). While the peak intensity is mostly unchanged in the ^1^H-amide-start spectrum, inspection of Figure 3B also clearly shows enhanced line broadening relative to the ^13^C-start experiment. Thus, while the integrated peak intensity is enhanced, relaxation losses during the additional spin echoes (especially for the ^1^H-methyl-start spectrum), and those resulting from excitation of the ^1^H-nucleus, offset the intended gains. This observation is consistent with our experiences using ^1^H-start strategies for backbone detection, where the expected signal enhancement is on occasion lost to efficient relaxation. For other protein systems, it remains possible that ^1^H-start CON-Kac spectroscopy will yield benefits. Despite the lack of sensitivity improvement realized here, these ^1^H-start experiments may also prove advantageous to future methods development as the starting points for 3D spectroscopy that aims to characterize the structure and dynamics of acetyllysine residues more thoroughly.

Maximal spectral resolution, generally limited by the number of points collected in the indirect dimension of a 2D spectrum, is imperative because lysine- Cε chemical shifts fall in a relatively narrow range (Ulrich et al., 2008). Non-uniform sampling (NUS) can theoretically be used to improve spectral resolution in this case, as one can reconstruct a higher number of points in the indirect dimension in an experiment using NUS compared with a traditionally sampled experiment collected in the same amount of time. Although we do not report NUS data here, the utility of this strategy has been reported for other ^13^C-detect experiments, including those utilizing the IPAP virtual decoupling scheme (Bermel et al., 2009; Bastidas et al., 2015). Coupled with the inherent narrow linewidth in the ^13^C direct dimension, this may present an opportunity to resolve resonances that are partially overlapped. However, care must be taken if NUS is implemented for real-time kinetics or spin relaxation measurements, as it is known that NUS can lead to intensity artifacts dependent on the sampling scheme chosen (Maciejewski et al., 2011).

**FIGURE 4:**
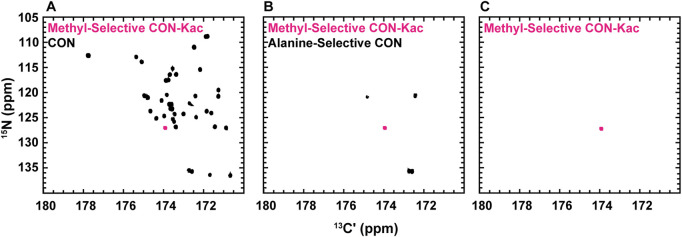
Spin-state selective [^13^Cʹ,^15^N] CON-Kac spectroscopy simplifies acetyllysine spectra in the background of uniform ^13^C- and ^15^N-labeling of the modified protein. **(A)** The backbone-optimized [^13^Cʹ,^15^N] CON displays excellent spectral dispersion and supports resonance assignment efforts. Overlay of the [^13^Cʹ,^15^N] CON-Kac indicates the position of the acetyllsine resonance, which is still present in the backbone-optimized experiment. **(B)** ^13^C direct-detect amino acid selective spectroscopy has been used to generate an alanine-selective [^13^Cʹ,^15^N] CON. Similarity in the spin topology of the acetamide functional group within acetyllysine and the alanine backbone results in both contributing to the overall spectrum. Overlay of the [^13^Cʹ,^15^N] CON-Kac indicates the position of the acetyllsine resonance for clarity. **(C)** The methyl-selective [^13^Cʹ,^15^N] CON-Kac, which employs the triple quantum filter from the alanine-selective CON, results from optimization to suppress the alanine resonances. The spectrum displayed was collected on ^13^C-acetylated, uniformly ^13^C, ^15^N-labeled H3 (1-44) and demonstrates excellent spectral simplification to unambiguously display the acetyllysine signal.

While the experiments described to this point require little to no isotopic enrichment of the target protein, it remains likely that, in many cases, investigators will want or need to produce uniformly ^13^C-, ^15^N-enriched protein samples, which may be valuable to acetylate. Interestingly, backbone-optimized [^13^C,^15^N] CON spectra do also yield resonances for the N_ε_ acetyllysine moiety, thus complicating spectral analysis (Figure 4A). Most importantly for the present application, the acetamide functional group differs in covalent structure from the polypeptide backbone in that the carbonyl is adjacent to a methyl carbon, as opposed to the α-carbon encountered in the backbone. Methyl groups can be selected for during the preparation period of a pulse sequence based on their spin topology granting access to a unique triple-quantum state, at a known cost of reduced sensitivity (Schubert et al., 1999). This property has previously been utilized to generate alanine-selective ^13^C direct-detect CON spectroscopy (Bermel et al., 2012; Sahu et al., 2014). Application of the alanine-selective pulse sequence to ^13^C-acetyl-labled H3 results in retention of both the alanine backbone resonances and the aceteyllysine (Figure 4B), likely because the transfer step from the methyl carbon to the carbonyl carbon refocuses both a one-bond and a long-range coupling. Indeed, further optimization to yield the methyl-selective [^13^C,^13^C] CaliCO-Kac suppresses the two-bond coupling from the alanine methyl to the backbone carbonyl. This results in a clean spectrum, presenting only the acetyllysine resonance, even when a uniformly ^15^N, ^13^C-enriched protein is used (Figure 4C). This strategy supports straightforward assignment of the acetamide resonance in a double-labelled sample generated for backbone chemical shift assignment and/or structure characterization, without the need to generate an additional NMR sample.

**FIGURE 5:**
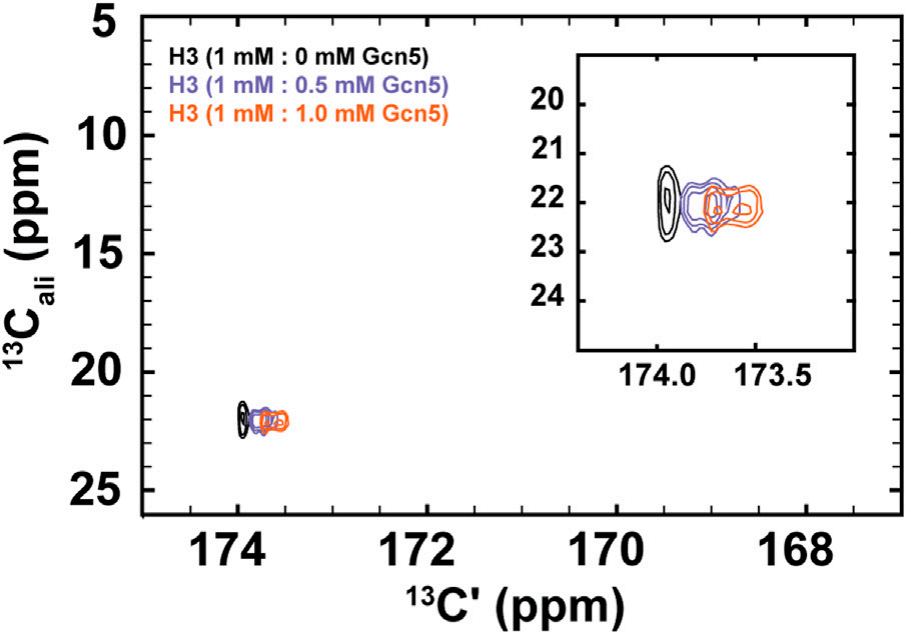
^1^H-start [^13^Cʹ,^13^C^ali^] CaliCO-Kac reveals chemical shift perturbations of the histone H3 acetyllsine resonance upon binding to the Gcn5 bromodomain. The spectrum of 1.0 mM ^13^C-acetyl H3 (1–44W) displays one resonance for the K14Ac/K18Ac (black). Addition of .5 mM bromodomain (purple) or 1.0 mM bromodomain (orange) results in resolution of the acetyllysine peaks into two distinct resonances, both of which show chemical shift perturbation in the carbonyl dimension, relative to the unbound state.

To demonstrate the biological utility of the methods described here, we have conducted NMR experiments that monitor Gcn5 bromodomain binding to acetylated histone H3. While bromodomains have a highly conserved structure, their acetyllysine binding pockets are lined with divergent amino acids across homologs, which may contribute to binding specificity. Additionally, bromodomain and extraterminal-domain (BET) family proteins are of interest for their application as pharmaceutical targets (Mujtaba et al., 2007; Cheung et al., 2021). Our results, presented in Figure 5, demonstrate the general potential for the methods described here to monitor binding and bound-state structure for bromodomain-acetyllysine interactions.

For these experiments, ^1^H-methyl-start [^13^C,^13^C] CaliCO-Kac spectra were collected 1 mM ^13^C-H3 (1–44W), titrated with either .5 mM or 1.0 mM natural isotope abundance Gcn5 bromodomain. While the chemical shift of the methyl carbon remains unchanged within the resolution of the experiment, a clear chemical shift perturbation in the carbonyl chemical shift is observed upon overlay of the spectra (Figure 5). In the presence of sub-stoichiometric Gcn5, two resonances are observed, neither of which directly overlay with the unbound resonance position. Additional chemical shift perturbations are observed at equimolar concentrations. These data suggest that the binding interaction is in fast exchange on the NMR timescale. Intriguingly, the presence of two resonances in the Gcn5-bound spectra suggests that K14Ac and K18Ac may have distinct carbonyl chemical shifts in the bound state, although this hypothesis must be followed up with detailed future studies. The clear conclusion from these data is that ^13^C direct-detect methods yield chemical shift perturbations upon change of biophysical state and are well-suited to studying acetyllsine interactions with binding partners.

## 4 Conclusion

Lysine N_ε_ acetylation is a prevalent protein post-translational modification, but the lack of a convenient and label-free method for its biochemical and structural characterization has hindered development of molecular scale models for its function. Here, we demonstrate that the synthesis of ^13^C-acetyl CoA for enzymatic acetylation of lysine residues in proteins is a facile approach to generating samples suitable for NMR spectroscopy. Further, we report the optimization of seven pulse programs that directly detect the acetyllysine mark, yielding easily interpreted spectra. Most importantly, the samples required are virtually unmodified, with the addition of only 2–3 Da of mass and no extrinsic atoms, which supports application of this method to investigation of modified protein structure, interactions, and enzymatic deacetylation in a variety of biologically relevant contexts.

Synthesis of ^13^C-acetyl CoA can be accomplished through a synthetic or enzymatic route. The synthetic route performs best in a two-step workflow where the acetyl-CoA is fully synthesized prior to enzymatic acetylation of a target protein substrate. The synthetic route activates all available CoASH to acetyl-CoA but is only able to utilize half of the ^13^C-acetate functionalities present in the anhydride starting material, discarding the other half as ^13^C acetate (although this can be recycled). In contrast, ACS is able to scavenge ^13^C-acetate to activate CoASH and produce ^13^C-acetyl CoA. Further, this enzyme functions best when coupled in a one-step workflow where ^13^C-acetyl CoA synthesis and enzymatic acetylation of the target protein occur simultaneously. To assay diverse lysine acetyltransferases (other than Ada2/Gcn5), some optimization of solution conditions may be required if, for example, the acetyltransferase of interest displays poor solubility or activity under solution conditions that favor ACS activity. ^13^C-acetate is a significantly more cost-effective starting material than ^13^C-acetic anhydride, and the enzymatic route uses a larger fraction of available ^13^C atoms in the ^13^C-acetyl CoA product. Thus, the fully enzymatic route to protein ^13^C-acetylation can be highly efficient.

Traditional [^1^H,^13^C]-HSQC spectroscopy is suitable to detect ^13^C-enriched acetyllysine but does have drawbacks compared to the new ^13^C direct-detect methods reported here. Specifically, disambiguation of the acetyllysine resonance from other peaks in the spectrum can be complicated. For example, the resonance from unincorporated acetate will remain in the spectrum, if it is not removed through buffer exchange prior to spectroscopy. Such a buffer exchange step would not be possible in the context of real time NMR monitoring of catalysis, which we have previously demonstrated to yield significant mechanistic insights for both serine phosphorylation (Gibbs et al., 2017) and lysine methylation (Usher et al., 2021). Further, many IDPs require organic co-solutes for solubility, stability, and to maintain a monomeric assembly state; in ^1^H-detected spectroscopy, these co-solvents will often introduce additional resonances that may create baseline distortions if present in sufficiently high concentration. ^13^C direct-detect methods are highly resilient to co-solute addition.

The methods described here rely on preparation of a suitable acetyltransferase to generate the NMR sample, but they do not oblige the investigator to recombinantly prepare target proteins with isotopic enrichment. So long as ^13^C-enriched acetate is transferred, either [^1^H,^13^C]-HSQC or [^13^C,^13^C] CaliCO-Kac spectroscopy can be employed on target protein from nearly any desired source. When isotopic enrichment is a possibility, [^13^C,^15^N] CON-Kac spectroscopy becomes available, yielding additional advantages. Specifically, because the detected carbon-nitrogen bond only exists in the acetylated lysine residue, and not in any precursor or biproduct of the reaction, its detection provides unambiguous evidence toward the installation of the acetyl group. Furthermore, the complexity of the selected spin system confers extremely high background suppression, which should support investigation of acetyllysine in the native nucleosome context, or even in the presence of cellular extracts.

In summary, we envision the biosynthetic and NMR spectroscopic techniques described here providing a simple, cost-effective, and versatile probe to study lysine acetylation *in vitro* in a broad range of contexts. Notably, these methods generalize well to any pairing of acetyltransferase enzyme and protein substrate; the selection of histone H3 and Ada2/Gcn5 was not required. If non-enzymatic means to install ^13^C-enriched acetate are employed, the NMR spectra described here will still provide an excellent means to characterize the resulting system. Alternatively, the enzymatic approaches described here also lend themselves well to preparation of isotopically enriched substrates for readout by mass spectrometry. Beyond monitoring the installation of acetyllysine modifications, we envision these techniques being used to monitor changes in the chemical environment of acetyllysine upon interaction with reader proteins, as demonstrated here through binding to the Gcn5 bromodomain, and for (potentially real-time) tracking of deacetylase activity. In the future, these methods can also be generalized to yield 3D spectroscopy to support the functional assays described here. Ultimately, wide use of these techniques should facilitate closure of the knowledge gap that currently exists between the list of known acetylated proteins and mechanistic insights into their functions.

## Data Availability

The datasets presented in this study can be found in online repositories. The names of the repository/repositories and accession number(s) can be found below: https://scholarsphere.psu.edu/resources/734c341d-8638-414b-909c-45f2bf6575b1.
