# Supplementary material for: A direct nuclear magnetic resonance method to investigate lysine acetylation of intrinsically disordered proteins
Source: Front Mol Biosci. 2023 Jan 6;9:1074743. doi: 10.3389/fmolb.2022.1074743 (PMC9853081; doi:10.3389/fmolb.2022.1074743)
Supplement: Supplementary file 1 [file DataSheet1.PDF]

frontiers

Supplementary Material

A Direct Nuclear Magnetic Resonance Method to Investigate Lysine Acetylation of Intrinsically Disordered Proteins

Olivia A. Fraser[1], Sophia M. Dewing[1], Emery T. Usher[1], Christy George[2], and Scott A. Showalter[1,2] *

[1]Center for Eukaryotic Gene Regulation, Department of Biochemistry and Molecular Biology, The Pennsylvania State University, University Park, PA 16802

[2]Department of Chemistry, The Pennsylvania State University, University Park, PA 16802

1 Supplementary Figures and Tables

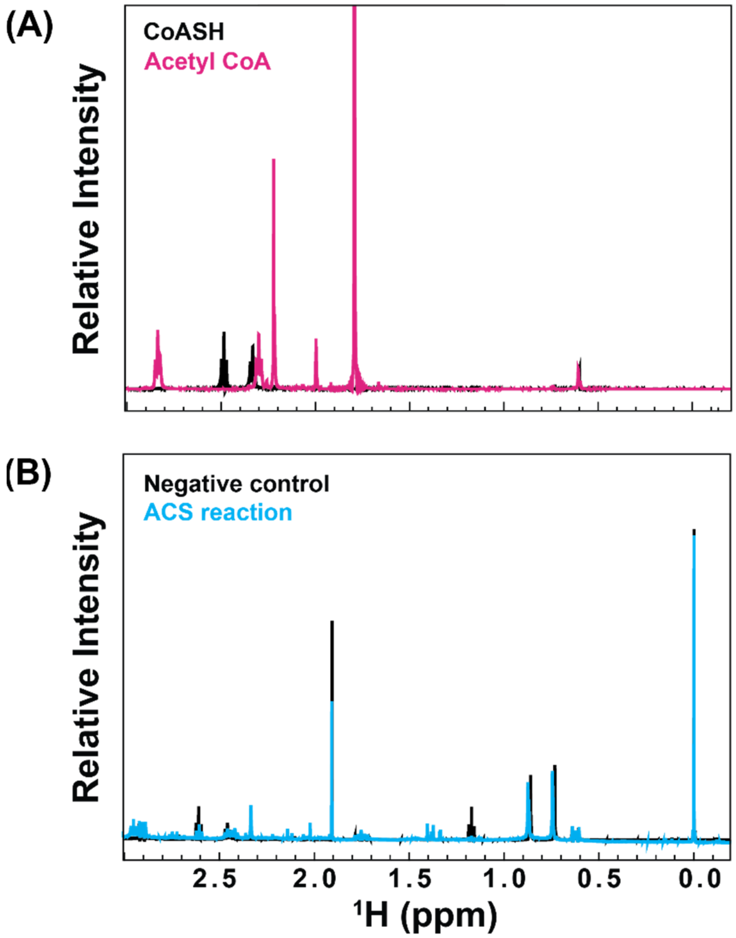

Figure S1. Characterization of the products from synthetic and enzymatic production of acetyl-coA. (A) 1D proton NMR spectrum of synthetically derived acetyl-CoA is shown in pink and CoASH starting material in black. The new peak that emerges at 2.22 ppm ^{1}H represents the methyl group of the acetyl moiety in the context of acetyl-CoA. Additionally, the methylene group adjacent to the sulfhydryl of CoASH shifts from 2.50 ppm ^{1}H in black to 2.83 ppm ^{1}H in the pink spectrum, consistent with published CoASH (https://coalabio.com/coenzyme-a-nmr-spectrum.html) and acetyl-CoA (https://coalabio.com/acetyl-coa-nmr-spectrum.html) spectra. (B) 1D proton NMR spectrum of enzymatically derived acetyl-CoA is shown in blue and CoASH starting material in black (blue). Inclusion of DSS internal standard allowed for quantitation of 36% acetyl-CoA yield.

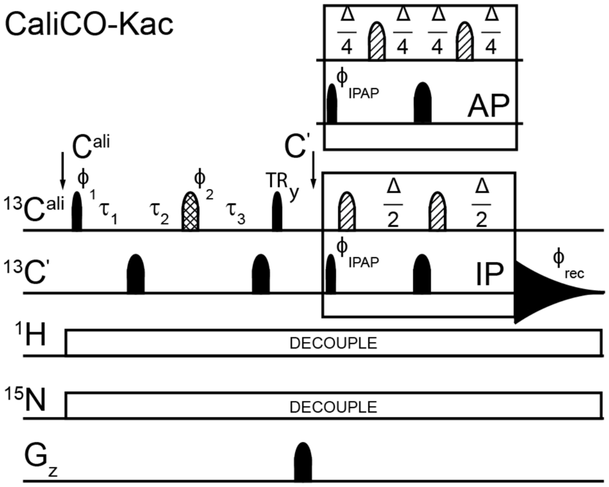

Figure S2. Pulse sequence for the carbon start [$^{13}C'$,$^{13}C^{ali}$]-CaliCO-Kac experiment. Band-selective pulses on the ^{13}C channel are presented on two logical channels with the respective frequencies indicated for clarity. All ^{13}C pulses are represented with narrow (90°, Q5_sebop) shapes and wide (180°, Q3_surbop) shapes. The pulses filled with diagonal lines are off-resonance 180° Q3_surbop shaped pulses centered at 25 ppm. The pulse filled with hashed lines is a higher selectivity 180° Q3_surbop shaped pulse centered at 25 ppm. The 'TR' label indicates a time-reversed 90° pulse. All pulses are applied with x-phase unless otherwise indicated. Pulsed field gradients (Gz) are also represented by shapes. Nitrogen decoupling is achieved with garp4 (220 µs, 2.367 W), and proton decoupling is achieved using waltz65 (70 µs, 0.096 W). The delay time Δ = 9.0 ms. $^{13}C^{ali}$ chemical shift evolution is measured during t_1 using a semi-constant time period with delays $\tau_1 = (\Delta + t_1)/2$, $\tau_2 = (1\text{-}\Delta/t_{1max})t_1/2$, and $\tau_3 = (1\text{-}t_1/t_{1max})\Delta/2$, where t_{1max} is the maximum duration of the t_1 labeling period. The phase cycle is φ_1 = x, -x; φ_2 = 4(x), 4(-x); φ_{IPAP}(IP) = x, x, -x, -x; φ_{IPAP}(AP) = -y, -y, y, y; φ_{REC} = x, -x, -x, x, -x, x, x, -x. Quadrature detection in the indirect dimension is obtained by States-TPPI incrementation of φ_1.

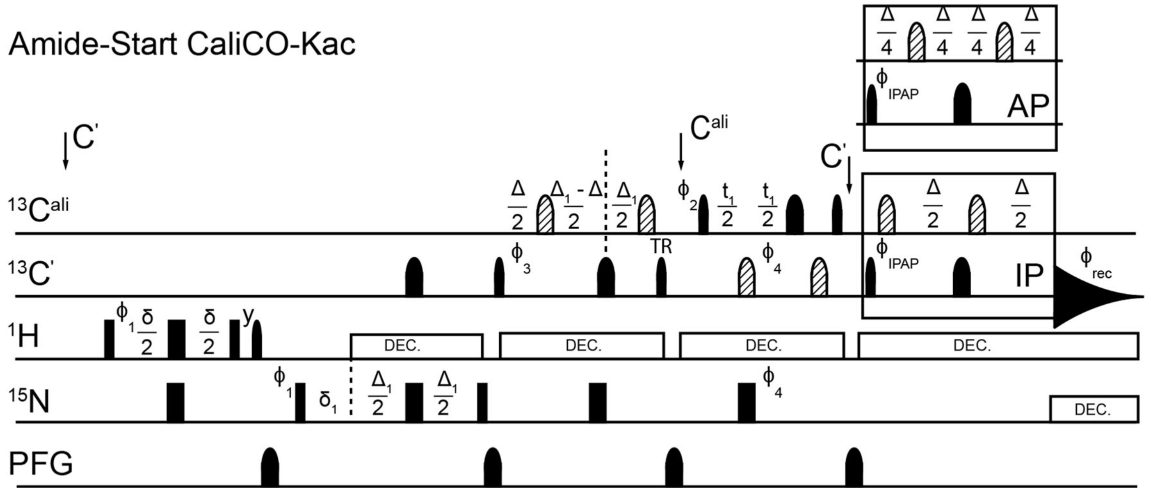

Figure S3. Pulse sequence for the amide-start [^{13}C′,^{13}C^{ali}]-CaliCO-Kac experiment. Band-selective pulses on the ^{13}C channel are presented on two logical channels with the respective frequencies indicated for clarity. The carrier position switched between ^{13}C′, centered at 172 ppm, and ^{13}C^{ali}, centered at 25 ppm, at the times indicated by vertical arrows. Narrow and wide rectangular pulses correspond to 90° and 180° hard pulses, respectively. All ^{13}C pulses are represented with narrow (90°, Q5_sebop) shapes and wide (180°, Q3_surbop) shapes. The pulses filled with diagonal lines are off-resonance 180° Q3_surbop shaped pulses. The 'TR' label indicates a time-reversed 90° pulse. All pulses are applied with x-phase unless otherwise indicated. The ^{1}H shaped pulse is water selective. Pulsed field gradients (Gz) are also represented by shapes. Nitrogen decoupling is achieved with garp4 (220 µs, 2.367 W), and proton decoupling is achieved using waltz65 (70 µs, 0.096 W). The delay times are δ = 4.6 ms, δ_1 = 5.5 ms, Δ = 9.8 ms, and Δ_1 = 25 ms. ^{13}C^{ali} chemical shift evolution is measured during t_1. The phase cycle is φ_1 = x, -x; φ_2 = x, x, -x, -x; φ_3 = 4(x), 4(-x); φ_4 = 8(x), 8(-x); φ_{IPAP}(IP) = x; φ_{IPAP}(AP) = -y; φ_{REC} = x, -x, -x, x, -x, x, x, -x. Quadrature detection in the indirect dimension is obtained by States-TPPI incrementation of φ_2.

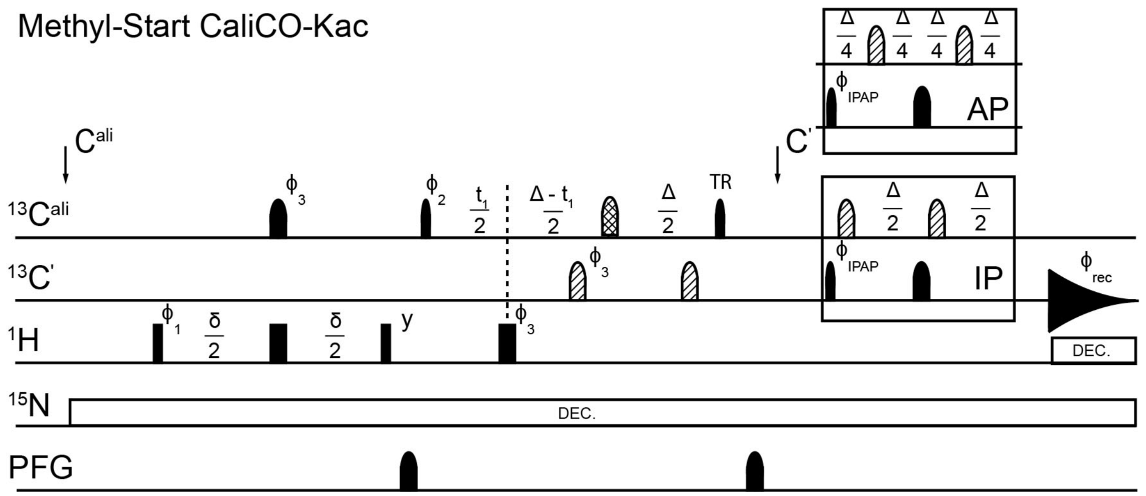

Figure S4. Pulse sequence for the methyl-start [$^{13}C'$,$^{13}C^{ali}$]-CaliCO-Kac experiment. Band-selective pulses on the ^{13}C channel are presented on two logical channels with the respective frequencies indicated for clarity. The carrier position switched between $^{13}C'$, centered at 172 ppm, and $^{13}C^{ali}$, centered at 25 ppm, at the times indicated by vertical arrows. Narrow and wide rectangular pulses correspond to 90° and 180° hard pulses, respectively. All ^{13}C pulses are represented with narrow (90°, Q5_sebop) shapes and wide (180°, Q3_surbop) shapes. The pulses filled with diagonal lines are off-resonance 180° Q3_surbop shaped pulses. The pulse filled with hashed lines is a higher selectivity 180° Q3_surbop shaped pulse centered at 25 ppm. The 'TR' label indicates a time-reversed 90° pulse. All pulses are applied with x-phase unless otherwise indicated. Pulsed field gradients (Gz) are also represented by shapes. Nitrogen decoupling is achieved with garp4 (220 µs, 2.367 W), and proton decoupling is achieved using waltz65 (70 µs, 0.096 W). The delay times are $\delta = 3.6$ ms, $\Delta = 9.0$ ms, and the initial value of $t_1 = 0.72$ ms. $^{13}C^{ali}$ chemical shift evolution is measured during t_1. The phase cycle is φ_1 = x, -x; φ_2 = x, x, -x, -x; φ_3 = 8(x), 8(-x); φ_{IPAP}(IP) = 4(x), 4(-x); φ_{IPAP}(AP) = 4(-y), 4(y); φ_{REC} = x, -x, -x, x, -x, x, x, -x. Quadrature detection in the indirect dimension is obtained by States-TPPI incrementation of φ_2.

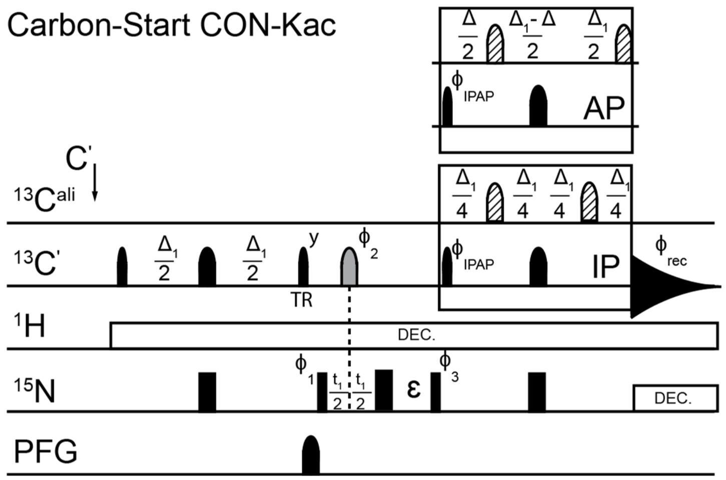

Figure S5. Pulse sequence for the carbon-start [^{13}C′,^{15}N]-CON-Kac experiment. Band-selective pulses on the ^{13}C channel are presented on two logical channels with the respective frequencies indicated for clarity. The carrier position is set to ^{13}C′, centered at 172 ppm. Narrow and wide rectangular pulses correspond to 90° and 180° hard pulses, respectively. All ^{13}C pulses are represented with narrow (90°, Q5_sebop) shapes and wide (180°, Q3_surbop) shapes. The pulses filled with diagonal lines are off-resonance 180° Q3_surbop shaped pulses. The pulse filled with hashed lines is a higher selectivity 180° Q3_surbop shaped pulse centered at 25 ppm. The gray pulse on ^{13}C indicates a band-selective ^{13}C′ and ^{13}C^{ali} inversion pulse. The 'TR' label indicates a time-reversed 90° pulse. All pulses are applied with x-phase unless otherwise indicated. Pulsed field gradients (Gz) are also represented by shapes. Nitrogen decoupling is achieved with garp4 (220 µs, 2.367 W), and proton decoupling is achieved using waltz65 (70 µs, 0.096 W). The delay times are Δ = 9.8 ms, Δ_1 = 25 ms, and $\varepsilon = t_1(0) + pC180$. ^{15}N chemical shift evolution is measured during t_1. The phase cycle is φ_1 = x, -x; φ_2 = x, x, -x, -x; φ_3 = 4(x), 4(-x); φ_{IPAP}(IP) = x; φ_{IPAP}(AP) = -y; φ_{REC} = x, -x, x, -x, -x, x, -x, x. Quadrature detection in the indirect dimension is obtained by States-TPPI incrementation of φ_3.

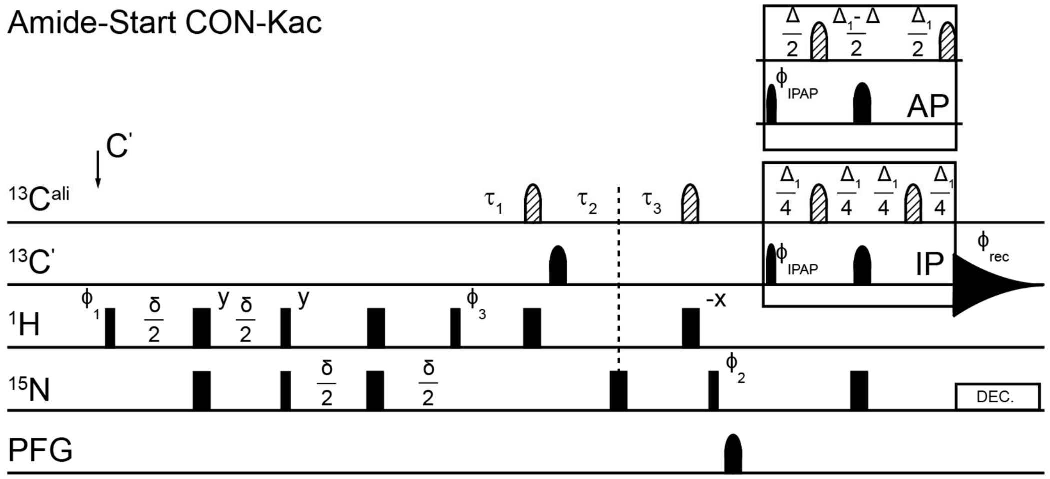

Figure S6. Pulse sequence for the carbon-start [$^{13}C'$,^{15}N]-CON-Kac experiment. Band-selective pulses on the ^{13}C channel are presented on two logical channels with the respective frequencies indicated for clarity. The carrier position is set to $^{13}C'$, centered at 172 ppm. Narrow and wide rectangular pulses correspond to 90° and 180° hard pulses, respectively. All ^{13}C pulses are represented with narrow (90°, Q5_sebop) shapes and wide (180°, Q3_surbop) shapes. The pulses filled with diagonal lines are off-resonance 180° Q3_surbop shaped pulses. All pulses are applied with x-phase unless otherwise indicated. Pulsed field gradients (Gz) are also represented by shapes. Nitrogen decoupling is achieved with garp4 (220 µs, 2.367 W). The delay times are δ = 5.4 ms, Δ = 9.8 ms, and Δ_1 = 25 ms. ^{15}N chemical shift evolution is measured during t_1 using a semi-constant time period with delays $\tau_1 = (\Delta_1 + t_1)/2$, $\tau_2 = (1-\Delta_1/t_{1max})t_1/2$, and $\tau_3 = (1-t_1/t_{1max})\Delta_1/2$, where t_{1max} is the maximum duration of the t_1 labeling period. The phase cycle is φ_1 = x, -x; φ_2 = x, x, -x, -x; φ_3 = -x, x; φ_{IPAP}(IP) = 4(x), 4(-x); φ_{IPAP}(AP) = 4(-y),4(y); φ_{REC} = x, -x,
-x, x, -x, x, x, -x. Quadrature detection in the indirect dimension is obtained by States-TPPI incrementation of φ_2.

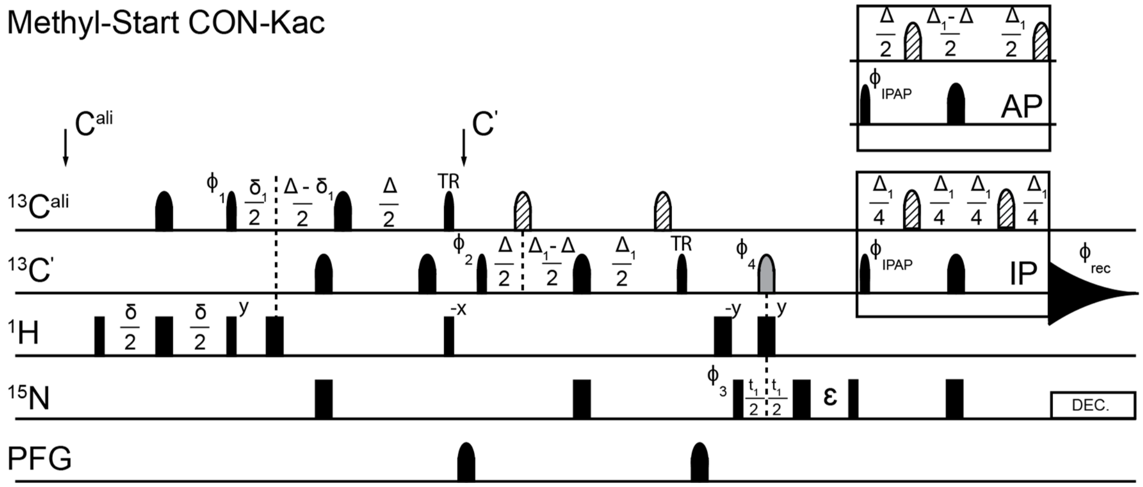

Figure S7. Pulse sequence for the methyl-start [^{13}C′,^{15}N]-CON-Kac experiment. Band-selective pulses on the ^{13}C channel are presented on two logical channels with the respective frequencies indicated for clarity. The carrier position switched between ^{13}C^{ali}, centered at 25 ppm, and ^{13}C′, centered at 172 ppm, at the times indicated by vertical arrows. Narrow and wide rectangular pulses correspond to 90° and 180° hard pulses, respectively. All ^{13}C pulses are represented with narrow (90°, Q5_sebop) shapes and wide (180°, Q3_surbop) shapes. The pulses filled with diagonal lines are off-resonance 180° Q3_surbop shaped pulses. The gray pulse on ^{13}C indicates a band-selective ^{13}C′ and ^{13}C^{ali} inversion pulse. The 'TR' label indicates a time-reversed 90° pulse. All pulses are applied with x-phase unless otherwise indicated. Pulsed field gradients (Gz) are also represented by shapes. Nitrogen decoupling is achieved with garp4 (220 µs, 2.367 W). The delay times are δ = 3.6 ms, δ_1 = 1.44 ms, Δ = 9.8 ms, Δ_1 = 25 ms, and $\varepsilon = t_1(0)$ + pC180. ^{15}N chemical shift evolution is measured during t_1. The phase cycle is φ_1 = 4(x), 4(-x); φ_2 = x, x, -x, -x; φ_3 = x, -x; φ_{IPAP}(IP) = x; φ_{IPAP}(AP) = -y; φ_{REC} = x, -x, -x, x, -x, x, x, -x. Quadrature detection in the indirect dimension is obtained by States-TPPI incrementation of φ_3.

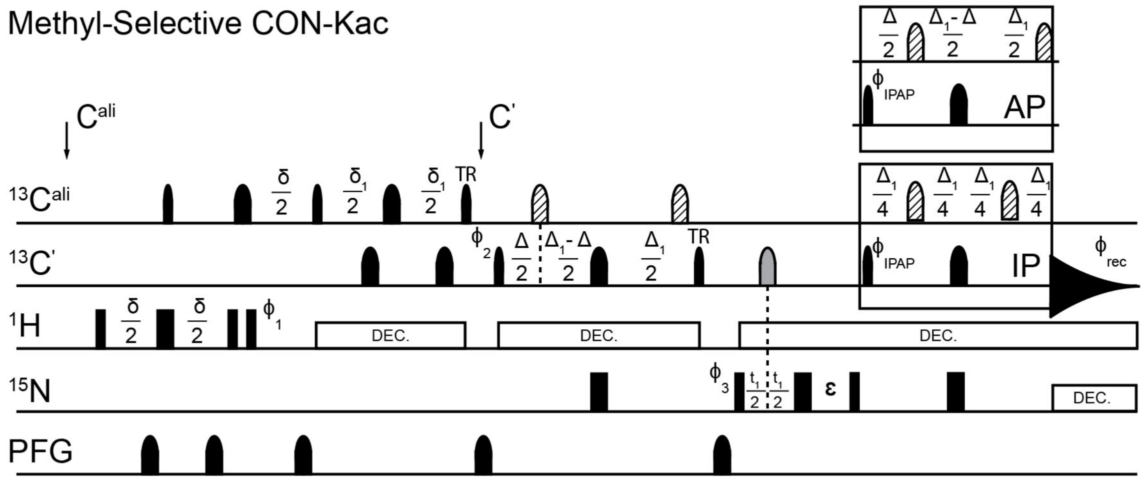

Figure S8. Pulse sequence for the methyl-selective d[^{13}C′,^{15}N]-CON-Kac experiment. Band-selective pulses on the ^{13}C channel are presented on two logical channels with the respective frequencies indicated for clarity. The carrier position switched between $^{13}C^{ali}$, centered at 25 ppm, and ^{13}C′, centered at 172 ppm, at the times indicated by vertical arrows. Narrow and wide rectangular pulses correspond to 90° and 180° hard pulses, respectively. All ^{13}C pulses are represented with narrow (90°, Q5_sebop) shapes and wide (180°, Q3_surbop) shapes. The pulses filled with diagonal lines are off-resonance 180° Q3_surbop shaped pulses. The gray pulse on ^{13}C indicates a band-selective ^{13}C′ and $^{13}C^{ali}$ inversion pulse. The 'TR' label indicates a time-reversed 90° pulse. All pulses are applied with x-phase unless otherwise indicated. Pulsed field gradients (Gz) are also represented by shapes. Nitrogen decoupling is achieved with garp4 (220 µs, 2.367 W), and proton decoupling is achieved using waltz65 (70 µs, 0.096 W). The delay times are δ = 8.0 ms, δ_1 = 5.0 ms, Δ = 9.0 ms, Δ_1 = 25 ms, and $\varepsilon = t_1(0)$ + pC180. ^{15}N chemical shift evolution is measured during t_1. The phase cycle is φ_1 = 2(30), 2(90), 2(150), 2(210), 2(270), 2(330); φ_2 = 12(x), 12(-x); φ_3 = x, -x; φ_{IPAP}(IP) = x; φ_{IPAP}(AP) = -y; φ_{REC} = 3(x, -x, -x, x), 3(-x, x, x, -x). Quadrature detection in the indirect dimension is obtained by States-TPPI incrementation of φ_3.
